# A Validated Age-Related Normative Model for Male Total Testosterone Shows Increasing Variance but No Decline after Age 40 Years

**DOI:** 10.1371/journal.pone.0109346

**Published:** 2014-10-08

**Authors:** Thomas W. Kelsey, Lucy Q. Li, Rod T. Mitchell, Ashley Whelan, Richard A. Anderson, W. Hamish B. Wallace

**Affiliations:** 1 School of Computer Science, University of St Andrews, St Andrews, United Kingdom; 2 School of Medicine, University of Edinburgh, Edinburgh, United Kingdom; 3 Royal Hospital for Sick Children, Edinburgh, United Kingdom; 4 MRC Centre for Reproductive Health, Queen’s Medical Research Institute, University of Edinburgh, Edinburgh, United Kingdom; 5 Department of Haematology/Oncology, Royal Hospital for Sick Children, Edinburgh, United Kingdom; Baylor College of Medicine, United States of America

## Abstract

The diagnosis of hypogonadism in human males includes identification of low serum testosterone levels, and hence there is an underlying assumption that normal ranges of testosterone for the healthy population are known for all ages. However, to our knowledge, no such reference model exists in the literature, and hence the availability of an applicable biochemical reference range would be helpful for the clinical assessment of hypogonadal men. In this study, using model selection and validation analysis of data identified and extracted from thirteen studies, we derive and validate a normative model of total testosterone across the lifespan in healthy men. We show that total testosterone peaks [mean (2.5–97.5 percentile)] at 15.4 (7.2–31.1) nmol/L at an average age of 19 years, and falls in the average case [mean (2.5–97.5 percentile)] to 13.0 (6.6–25.3) nmol/L by age 40 years, but we find no evidence for a further fall in mean total testosterone with increasing age through to old age. However we do show that there is an increased variation in total testosterone levels with advancing age after age 40 years. This model provides the age related reference ranges needed to support research and clinical decision making in males who have symptoms that may be due to hypogonadism.

## Introduction

In the male, testosterone secretion from the Leydig cells in the testes has a central role in developing secondary sexual characteristics, supporting spermatogenesis and regulating libido [1]. Synthesis and secretion are under the stimulation of the gonadotrophin luteinizing hormone (LH) from the anterior pituitary gland and approximately 98% of circulating testosterone is bound to plasma proteins, with the remaining 2% circulating freely [2]. Whether healthy adult men maintain serum testosterone concentrations throughout life, and the implications of a postulated decline and thus its potential for therapy, have been widely debated but remain unclear [3], [4].

A number of studies have reported decreasing testosterone levels in men with age. This includes a study involving both cross-sectional and longitudinal components [5], that reported low levels (<11.3 nmol/L) of total testosterone in up to 20% of men over 60, 30% over 70 and 50% over 80 years of age and suggested that further investigation of testosterone replacement in aged men, perhaps targeted to those with the lowest serum testosterone concentrations, was justified [5]. Longitudinal observation in the Massachusetts Male Aging Study also showed a decrease in total testosterone (TT) with increasing age [6], particularly when accompanied by increasing obesity. Male hypogonadism is a clinical condition resulting from testosterone deficiency as a result of abnormalities of testicular, hypothalamic or pituitary function. The diagnosis is based on clinical and biochemical findings and has been shown to be associated with impaired sexual function, impaired cognitive function [7], [8], depression [9], abdominal obesity, low bone and muscle mass [10], diabetes, and prediabetic states (including insulin resistance, impaired glucose tolerance and the metabolic syndrome) that may lead to an increase in risk of cardiovascular disease [11]–[13]. Overall, cardiovascular mortality is increased in late-onset hypogonadism [14], [15]. Testosterone replacement in young hypogonadal men results in significant improvement of libido and sexual function and is of clear benefit [16], but it remains to be established in older men that general health and other manifestations of the metabolic syndrome are improved [17]–[19]. Testosterone replacement therapy in older hypogonadal men is associated with an increased risk of cardiovascular events [20], [21], and such concerns have led to a current reappraisal of the safety of testosterone replacement (http://www.fda.gov/drugs/drugsafety/ucm383904.htm).

As the diagnosis of hypogonadism includes identification of serum testosterone levels below the normal range for healthy males, there is an underlying assumption that normal ranges of testosterone for the healthy population are known for all ages. However, to our knowledge, no such reference model exists in the literature, and hence the identification of individuals as hypogonadal does not have a widely applicable biochemical basis. In this study we derive and validate a normative model of TT across the lifespan.

## Methods

### Data acquisition

Using an established methodology [22]–[24], studies containing TT measurements of healthy human males at a known age were identified by performing Medline and Embase searches, using the search terms Humans, Testosterone, Males and Reference values. The reference lists of selected studies were checked, and cited references were retrieved to identify further relevant studies. Manuscripts were included for analysis if they reported TT levels for healthy normal males with no known testicular or endocrinological disorders. Data from subjects with an identified chronic illness or on testosterone replacement therapy were excluded from the dataset, as were data from fetal blood and cord blood. These studies provided the basis for a dataset for TT that approximates the healthy human population from childhood to old age. Twenty-seven studies were identified that met the inclusion criteria; eleven studies were excluded because only descriptive statistics for study data were reported [25]–[35].

For the remaining sixteen studies, data were extracted from scatterplots using Web Plot Digitizer v2.6 (http://arohatgi.info/WebPlotDigitizer) to convert the datapoints into pairs of numerical values denoting age and TT (n = 10,458; age range 0–101 years). Two researchers (TWK & LQL) extracted data from each plot with the results being compared (a) for inter-observer agreement and (b) agreement with the published descriptive statistics. Inter-observer agreement limits were set at 99% for both age and TT levels; in the event that this limit was not reached due to observer or calibration error, the data were extracted again by both observers. Longitudinal data were recorded as cross-sectional values. Serum testosterone values were standardised to give units of testosterone in nmol/L using the standard multiplication factor 1 ng/dL = 0.0347 nmol/L.

The current gold standard assay for male testosterone is mass spectroscopy-based LC-MS/MS [36], and several of our data sources measured testosterone using non-extraction platform immunoassays. A recent analysis of serum testosterone measurements (n = 3,174) comparing one such platform immunoassay demonstrated good accuracy at all concentrations found in eugonadal as well as hypogonadal men, when compared to mass spectroscopy assay values [37]. A further search of the biomedical literature was therefore performed to identify conversion formulae from other assay values to LC-MS/MS values [38]–[40], so that our modelling is concordant with current endocrinological best practice. Since the conversions used correlate strongly (85%–97%, Table 1) and have small Bland-Altman mean differences [41], their use introduces no significant bias into the combined data. For three of our identified studies [42]–[44], no such conversion formula was found (in-house assays were used and no direct comparison with LC-MS/MS values was made), and their data were therefore excluded. Data were also censored at age 3 years: extracted values below this age failed to match the descriptive statistics published with the chart, and therefore could not be used to model accurately the height or age of the expected peak in TT in early infancy.

**Table 1:** Total testosterone data summary.

Ref	1st Author	Assay	Conversion formula	r	Conversion source	*n*	Ages
[57]	Abdelrahaman	DPC	y = 1.098x−0.1	0.92–0.97	Wang et al.	47	6.2 (4.9–9.0)
[58]	Bergada	DSL	y = 1.791x−0.178	0.85	Scaglia y col.	94	0.02 (0–0.1)
[59]	Elmlinger	Centaur	y = 1.195*x−0.05	0.92–0.97	Wang et al.	623	40.1 (0–98.9)
[28]	Friedrich	Centaur	y = 1.195*x−0.05	0.92–0.97	Wang et al.	971	47.4 (20.3–79.8)
[60]	Kyriakopoulou	LS-MS/MS	NA	NA	NA	172	8.3 (0–18.6)
[61]	Leifke	DSL	y = 1.791x−0.178	0.85	Scaglia y col.	572	40.5 (20.0–80.0)
[5]	Mitchell Harman	DSL	y = 1.791x−0.178	0.85	Scaglia y col.	890	56.9 (22.9–94.4)
[49]	Morley	DPC	y = 1.098x−0.1	0.92–0.97	Wang et al.	287	76.2 (61.2–101.1)
[29]	Yeap	DPC	y = 1.098x−0.1	0.92–0.97	Wang et al.	3645	76.6 (70.8–87.8)
[62]	Pierik	Centaur	y = 1.195x−0.05	0.92–0.97	Wang et al.	113	0.2−(0.1–0.4)
[26]	Halmenschlager	Roche	y = 1.167x−2.62	0.92–0.97	Wang et al.	428	51.9 (29.8–83.0)
[63]	Sartorious	LS-MS/MS	NA	NA	NA	324	59.8 (40.1–86.9)
[6]	Travison	RIA	x = 0.706y^1.077^	0.96	Janse et al.	2194	61.5 (44.9–80.0)

The 13 papers listed were used as data sources, identified by reference number and first author. In the conversion formulae, x denotes the study assay and y denotes LS-MS/MS; r denotes the published correlation coefficient for the conversion formula. Median and range of ages are in years. NA denotes not applicable since no conversion is needed.

The final dataset (n = 10,097; age range 3–101 years) obtained from 13 studies (Table 1) represents a typical random sample from the healthy male population, and was used as the basis for normative model selection and verification. All data in this study were extracted from existing publications in the scientific and medical literature. Data relating to individual human subjects was not included and therefore specific ethical approval was not required.

### Data analysis

Zero TT values at conception were added to the combined dataset, in order to force models through the only known level at any age; these values were not taken into account when calculating model errors and fit. Since variability increases with testosterone level, the data were log-adjusted (after adding one to each value so that zero testosterone on a chart represents zero testosterone level). We then fitted 330 mathematical models to the data using TableCurve-2D (Systat Software Inc., San Jose, California, USA), and ranked the results by coefficient of determination, r^2^. Each model defines a generic type of curve and has parameters which, when instantiated gives a specific curve of that type. For each model we calculated values for the parameters that maximise the r^2^ coefficient. The Levenberg-Marquardt non-linear curve-fitting algorithm was used throughout, with convergence to 9 significant figures after a maximum of 4,000 iterations, for models having up to 21 parameters. For each candidate model, the mean square error and r^2^ were calculated after removing the artificial zero values at conception. In addition LOESS regression [45] was used to investigate the possibility that the best predictive model may be an ensemble of locally linear or quadratic models, rather than a single model covering all age ranges.

The best performing family of models were rational polynomials. 5-fold cross validation was performed: the data were randomly split into 5 equally sized subsets. For each subset S, the other four subsets were used to train rational polynomials having 3–11 parameters, with subset S being held back as test data. The mean square error of the test data was calculated and compared to the mean square error of training data for the same model. In other words, the estimated prediction error of a model when generalized to unseen data was compared to the training error of the model. A model was considered validated if

1. the residuals of the test data were approximately normally distributed, and
2. the tradeoff between high r2 for the training data (denoting possible overfitting to the data) and low generalisation error for the unseen test data (denoting possible underfitting to the data) was optimal.

## Results

**Figure 1:**
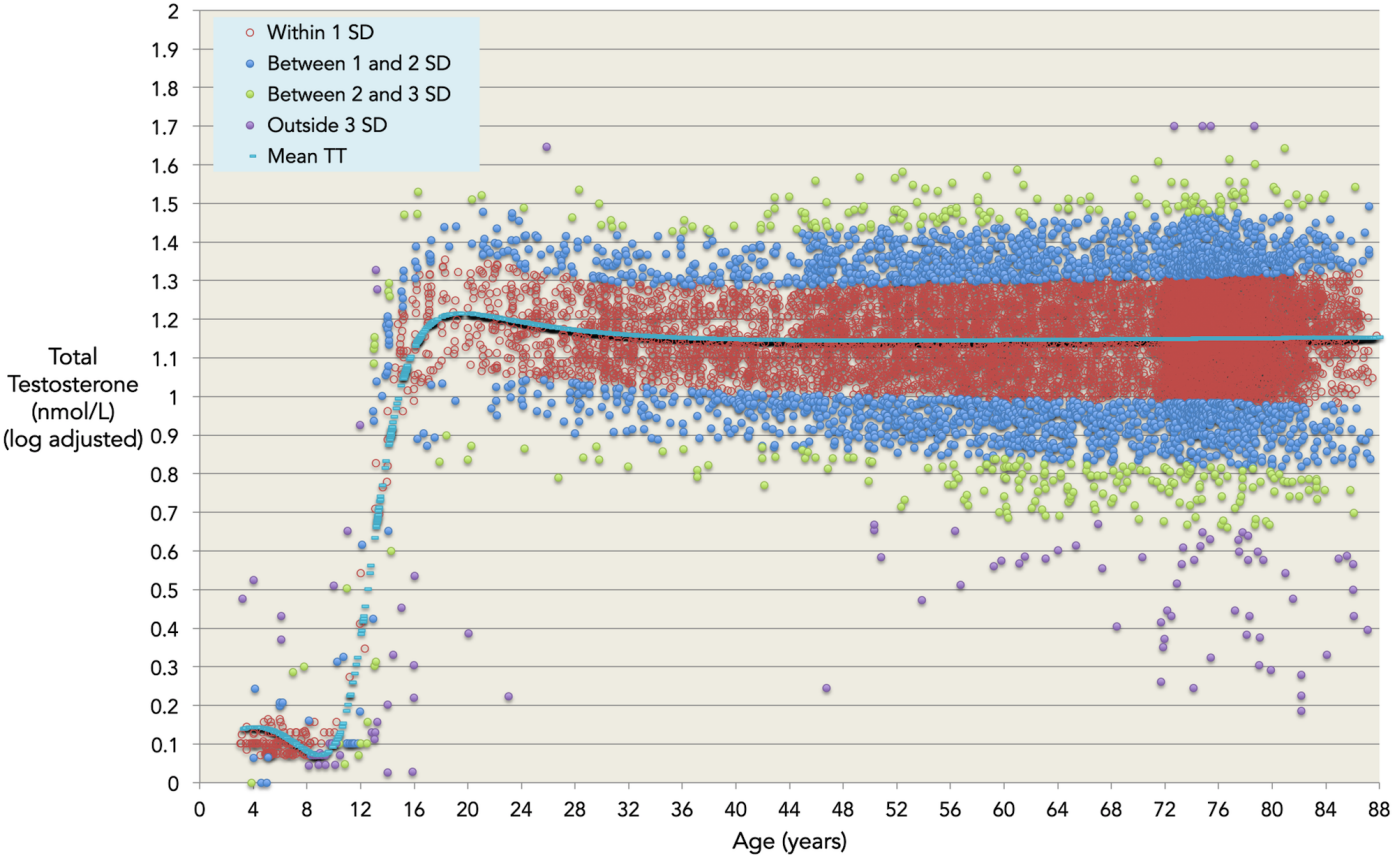
The validated model, log-adjusted testosterone values. Our dataset (n = 10,098) of log-adjusted observed total testosterone for ages 3–88 years, split into normative ranges determined by mean predicted values (blue line) and one (red), two (blue), three (green), and four (purple) standard deviations higher and lower than the predicted values.

**Figure 2:**
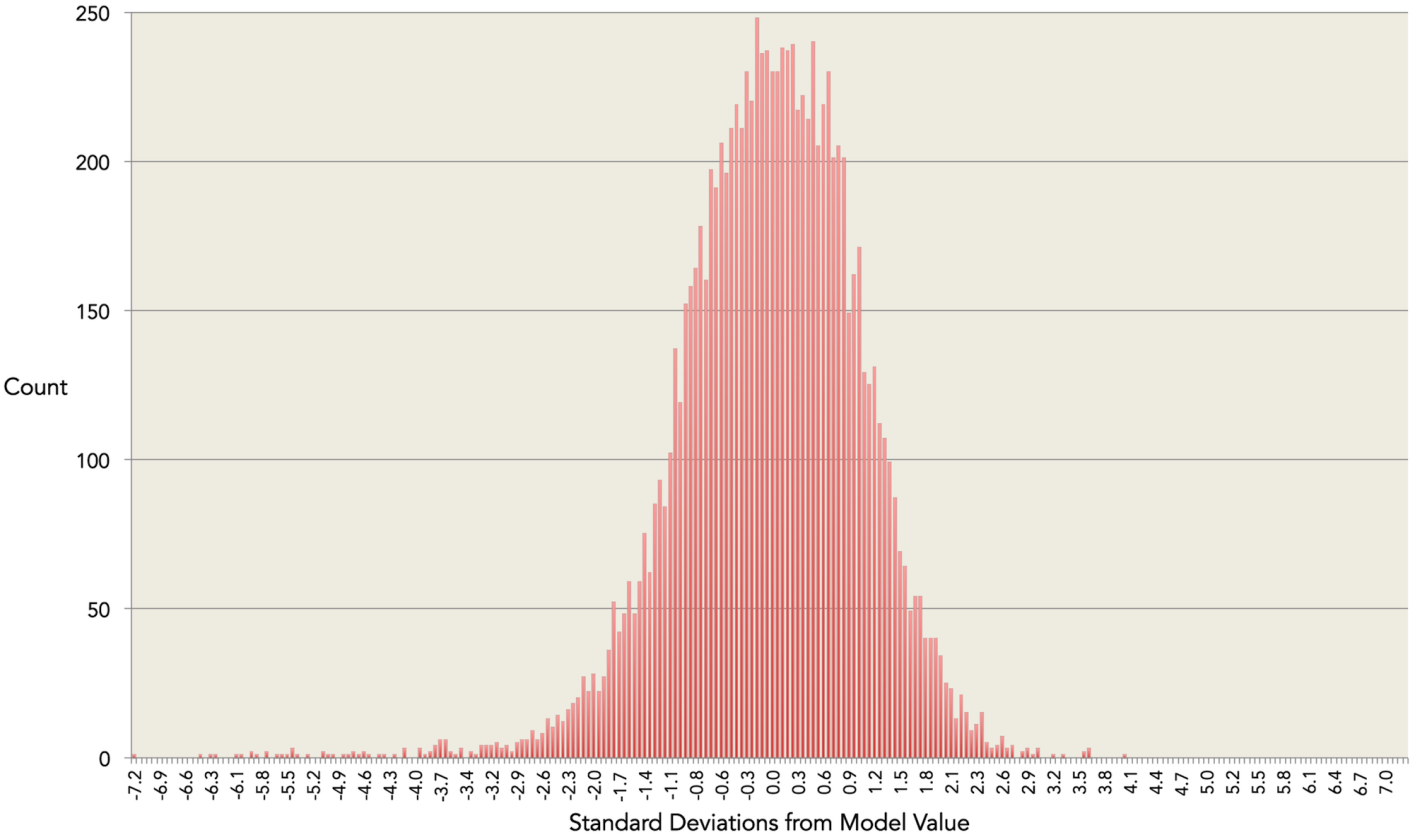
Residual analysis. The residuals are the variations in log-adjusted observed values from the log-adjusted age-related mean value predicted by the model. The residuals have excellent goodness of fit to an ideal Gaussian curve (r^2^ = 0.99). 71% of the residuals are within one standard deviations (SD) of the mean, 95% within 2 SD, and 99% within 3SD. The percentages for an ideal Gaussian distribution are 68%, 95% and 99% respectively.

**Figure 3:**
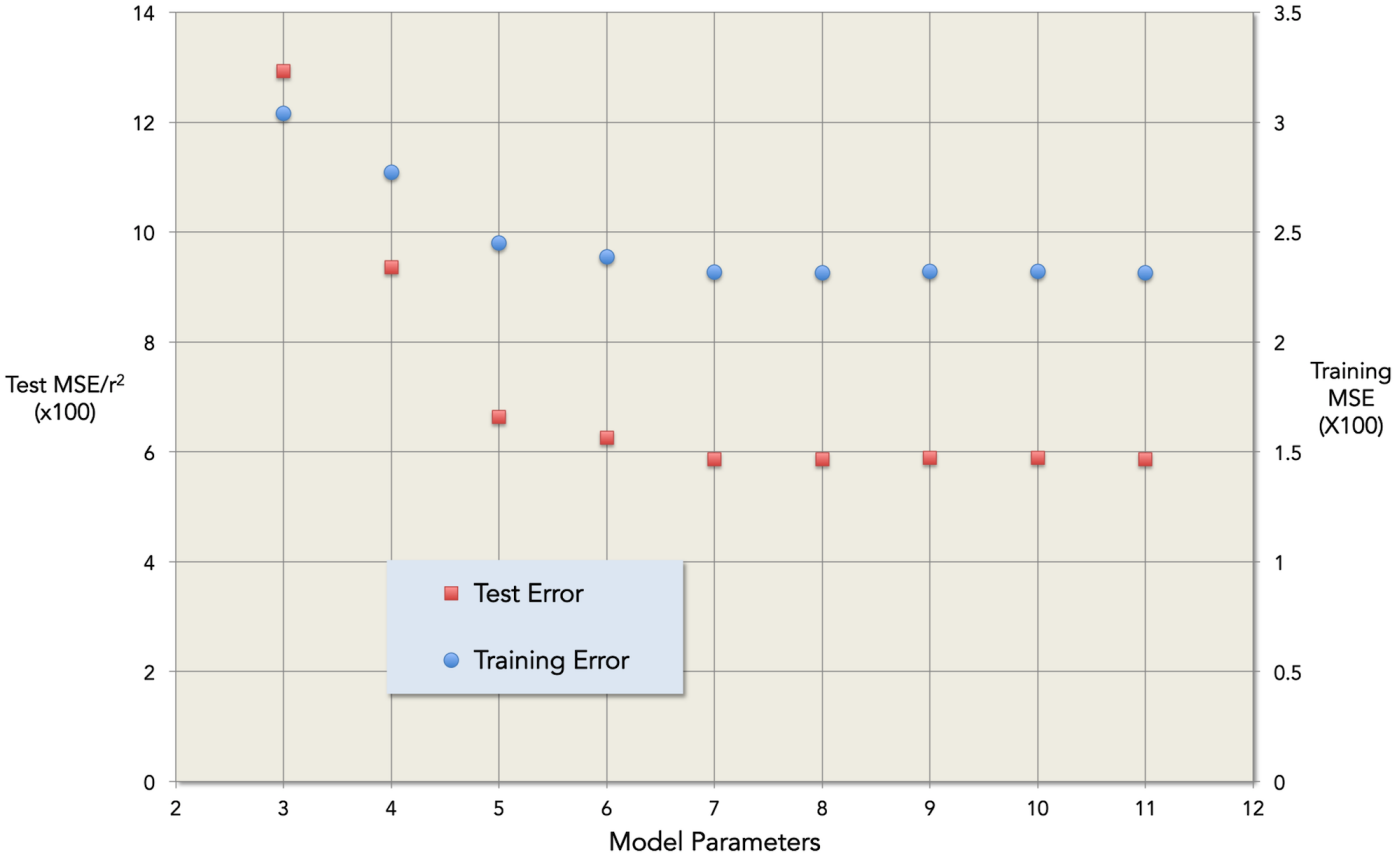
Exemplar of model validation stage. High test and training errors represent underfit (i.e. insufficient model parameters to accurately capture essential features of the dataset), and high test errors represent overfit (i.e. a model that will not generalise to accurately predict new data). The optimal number of model parameters is seven in this instance, and in the analysis of the four other cross validation sets.

The validated model is a rational polynomial of the form
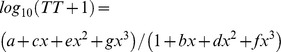
where TT is measured in nmol/L and x denotes age in years. Model coefficients a – f are given in Table 2, and relationship to the data given in Figure 1. The model has coefficient of determination r^2^ = 0.41 indicating that around 41% of the variation in serum TT throughout healthy male life is due to age alone, and that 59% in the variation is therefore due to other factors such as lifestyle, anthropometry and health status. The r^2^ for the best-fitting LOESS model was 0.32, establishing the optimality of the single regression model in terms of goodness-of-fit. The residual plot for the validated model (Figure 2) shows a distribution close to the ideal Gaussian curve (r^2^ = 0.99). Moreover, the proportions of residuals within one, two and three standard deviations (respectively 71%, 96% and 99%) are close to the expected values for data with a Gaussian distribution (respectively 68%, 95% and 99%). Figure 3 is an exemplar of the 5-fold validation process in which a model is chosen that neither overfits nor underfits the underlying dataset.

**Table 2:** Model parameter values.

Parameter	Value	Std Error	T Value	95% Conf Lim	95% Conf Lim2
a	0.04655	0.00718	6.48262	0.03247	0.06063
b	−0.05311	0.00748	−7.09759	−0.06777	−0.03844
c	0.05123	0.00629	8.15049	0.03891	0.06355
d	−0.00793	0.00142	−5.59626	−0.01070	−0.00515
e	−0.01222	0.00148	−8.25065	−0.01513	−0.00932
f	0.00058	0.00007	8.01130	0.00044	0.00072
g	0.00069	0.00008	8.49093	0.00053	0.00085

Also given are the standard error and T statistic for the values, and the upper and lower 95% confidence limits for the values.

**Figure 4:**
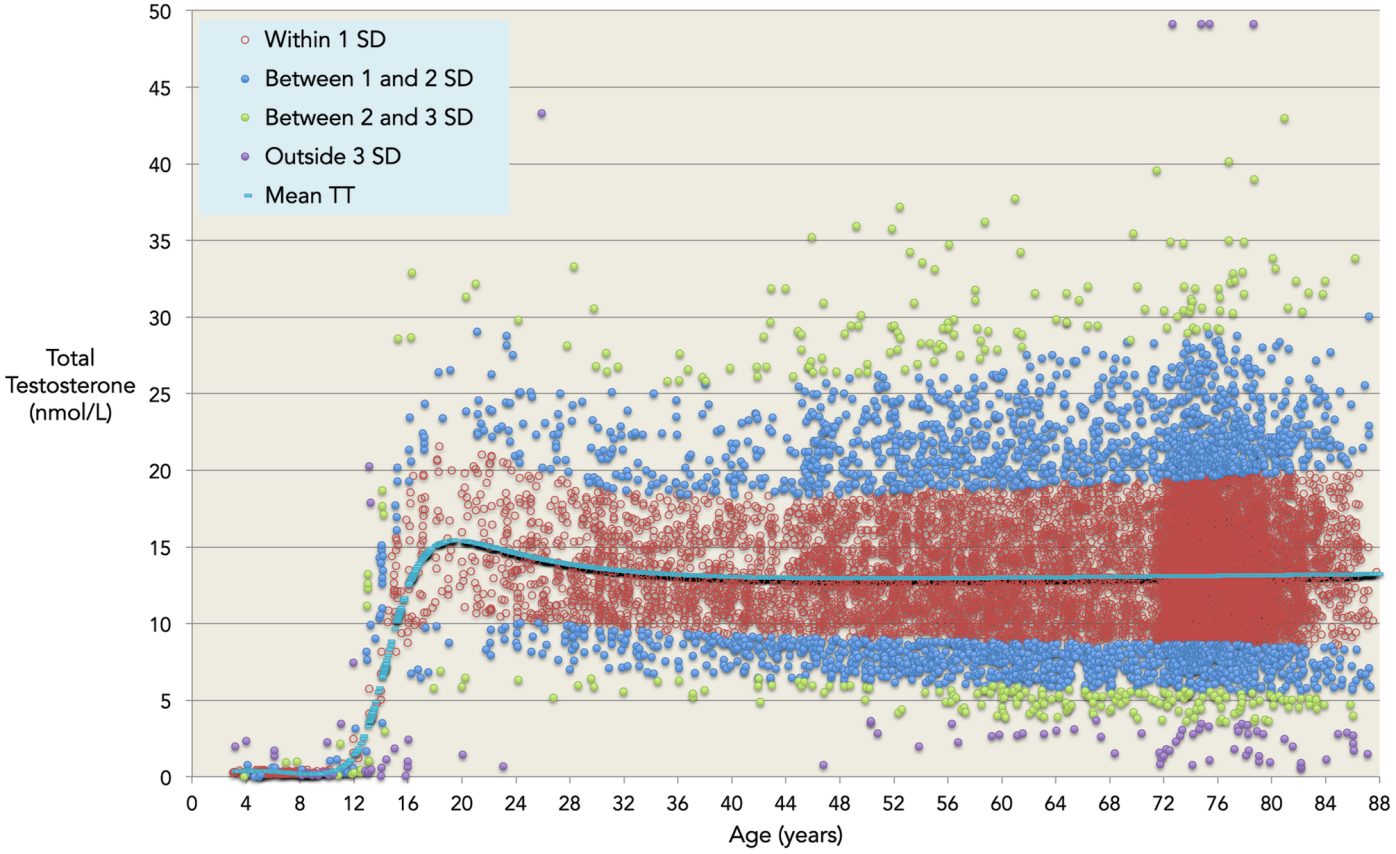
The validated model. Our dataset (n = 10,098) of observed total testosterone for ages 3–88 years, split into normative ranges determined by mean predicted values (blue line) and one (red), two (blue), three (green), and four (purple) standard deviations higher and lower than the predicted values.

**Figure 5:**
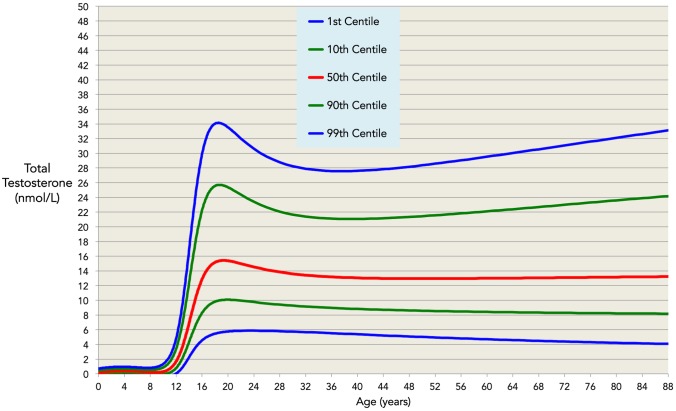
The validated model in centiles. Normative ranges for the model of total testosterone from ages 3–88 years. In the average case (red line) total testosterone remains constant for age >40. However, the variance in normative ranges increases for these ages, with 1^st^ to 99^th^ centile ranges of 5.6–27.6 nmol/L at age 35 years and 4.1–33.1 nmol/L at age 88 years.

Our log-unadjusted normative model (Figure 4) provides average TT values for the entire age range, together with normative ranges in terms of standard deviations away from age-related mean levels. The same model is given in terms of centiles in Figure 5. Residual plots for each decade of age are supplied as supporting information (Figure S1a–h), as are the remaining cross-validation plots (Figure S2), and the TableCurve inputs and output for the validated model (Table S1). Mean and normative ranges for serum TT in healthy males are given for ages from 3 to 88 years in Table 3.

**Table 3:** Normative age-related total testosterone reference values in nmol/L.

Age	1	2.5	10	20	30	40	50	60	70	80	90	97.5	99
3	0.0	0.0	0.1	0.2	0.3	0.3	0.4	0.4	0.5	0.6	0.7	0.9	1.0
4	0.0	0.0	0.1	0.2	0.3	0.3	0.4	0.5	0.5	0.6	0.7	0.9	1.0
5	0.0	0.0	0.1	0.2	0.3	0.3	0.4	0.4	0.5	0.6	0.7	0.8	0.9
6	0.0	0.0	0.1	0.2	0.2	0.3	0.3	0.4	0.4	0.5	0.6	0.8	0.9
7	0.0	0.0	0.0	0.1	0.2	0.2	0.3	0.3	0.4	0.5	0.6	0.7	0.8
8	0.0	0.0	0.0	0.1	0.1	0.2	0.2	0.3	0.3	0.4	0.5	0.7	0.8
9	0.0	0.0	0.0	0.0	0.1	0.1	0.2	0.3	0.4	0.5	0.6	0.8	0.9
10	0.0	0.0	0.0	0.1	0.2	0.2	0.3	0.4	0.5	0.7	0.9	1.2	1.3
11	0.0	0.0	0.2	0.3	0.5	0.6	0.7	0.9	1.1	1.3	1.6	2.1	2.4
12	0.0	0.3	0.8	1.1	1.3	1.5	1.7	2.0	2.4	2.8	3.4	4.3	4.8
13	0.9	1.4	2.2	2.7	3.1	3.5	3.8	4.4	5.1	5.9	7.0	8.8	9.7
14	2.3	3.0	4.4	5.2	5.9	6.4	6.9	8.0	9.2	10.5	12.4	15.3	16.9
15	3.6	4.6	6.6	7.9	8.8	9.5	10.3	11.8	13.4	15.4	18.0	22.1	24.3
16	4.6	5.9	8.3	9.8	11.0	11.9	12.9	14.7	16.7	19.0	22.2	27.2	29.9
17	5.1	6.6	9.3	11.1	12.3	13.4	14.4	16.4	18.6	21.1	24.6	30.0	32.9
18	5.5	7.0	9.8	11.7	13.0	14.1	15.2	17.2	19.4	22.0	25.6	31.1	34.0
19	5.6	7.2	10.0	11.9	13.2	14.3	15.4	17.4	19.6	22.2	25.7	31.1	34.1
20	5.8	7.3	10.1	11.9	13.2	14.3	15.4	17.4	19.5	21.9	25.4	30.7	33.5
21	5.8	7.3	10.0	11.8	13.1	14.2	15.2	17.1	19.2	21.6	24.9	30.0	32.8
22	5.9	7.3	10.0	11.7	12.9	14.0	15.0	16.8	18.8	21.2	24.4	29.3	32.0
23	5.9	7.3	9.9	11.5	12.8	13.8	14.8	16.6	18.5	20.7	23.9	28.7	31.3
24	5.9	7.2	9.8	11.4	12.6	13.6	14.5	16.3	18.2	20.4	23.4	28.1	30.6
25	5.9	7.2	9.7	11.3	12.4	13.4	14.3	16.0	17.9	20.0	23.0	27.6	30.0
26	5.8	7.2	9.6	11.1	12.3	13.2	14.1	15.8	17.6	19.7	22.6	27.1	29.5
27	5.8	7.1	9.5	11.0	12.1	13.1	14.0	15.6	17.4	19.5	22.3	26.7	29.1
28	5.8	7.1	9.4	10.9	12.0	13.0	13.8	15.5	17.2	19.2	22.1	26.4	28.8
29	5.8	7.0	9.3	10.8	11.9	12.8	13.7	15.3	17.0	19.1	21.8	26.2	28.5
30	5.7	7.0	9.3	10.8	11.8	12.7	13.6	15.2	16.9	18.9	21.7	25.9	28.2
31	5.7	6.9	9.2	10.7	11.7	12.7	13.5	15.1	16.8	18.8	21.5	25.8	28.0
32	5.7	6.9	9.1	10.6	11.7	12.6	13.4	15.0	16.7	18.7	21.4	25.6	27.9
33	5.6	6.8	9.1	10.6	11.6	12.5	13.3	14.9	16.6	18.6	21.3	25.5	27.8
34	5.6	6.8	9.0	10.5	11.5	12.4	13.3	14.9	16.5	18.5	21.2	25.4	27.7
35	5.6	6.8	9.0	10.5	11.5	12.4	13.2	14.8	16.5	18.4	21.2	25.4	27.6
36	5.5	6.7	9.0	10.4	11.5	12.3	13.2	14.7	16.4	18.4	21.1	25.3	27.6
37	5.5	6.7	8.9	10.4	11.4	12.3	13.1	14.7	16.4	18.4	21.1	25.3	27.6
38	5.4	6.7	8.9	10.3	11.4	12.3	13.1	14.7	16.4	18.3	21.1	25.3	27.6
39	5.4	6.6	8.9	10.3	11.3	12.2	13.1	14.7	16.3	18.3	21.1	25.3	27.6
40	5.4	6.6	8.8	10.3	11.3	12.2	13.0	14.6	16.3	18.3	21.1	25.3	27.6
41	5.3	6.5	8.8	10.2	11.3	12.2	13.0	14.6	16.3	18.3	21.1	25.3	27.7
42	5.3	6.5	8.8	10.2	11.3	12.2	13.0	14.6	16.3	18.3	21.1	25.4	27.7
43	5.3	6.5	8.7	10.2	11.3	12.2	13.0	14.6	16.3	18.3	21.1	25.4	27.8
44	5.2	6.4	8.7	10.2	11.2	12.1	13.0	14.6	16.3	18.4	21.2	25.5	27.8
45	5.2	6.4	8.7	10.2	11.2	12.1	13.0	14.6	16.3	18.4	21.2	25.6	27.9
46	5.1	6.4	8.7	10.1	11.2	12.1	13.0	14.6	16.4	18.4	21.2	25.6	28.0
47	5.1	6.3	8.6	10.1	11.2	12.1	13.0	14.6	16.4	18.4	21.3	25.7	28.1
48	5.1	6.3	8.6	10.1	11.2	12.1	13.0	14.6	16.4	18.5	21.3	25.8	28.2
49	5.0	6.3	8.6	10.1	11.2	12.1	13.0	14.6	16.4	18.5	21.4	25.9	28.3
50	5.0	6.3	8.6	10.1	11.2	12.1	13.0	14.6	16.4	18.5	21.4	25.9	28.4
51	5.0	6.2	8.6	10.1	11.2	12.1	13.0	14.6	16.5	18.6	21.5	26.0	28.5
52	4.9	6.2	8.5	10.1	11.1	12.1	13.0	14.7	16.5	18.6	21.6	26.1	28.6
53	4.9	6.2	8.5	10.0	11.1	12.1	13.0	14.7	16.5	18.6	21.6	26.2	28.7
54	4.9	6.2	8.5	10.0	11.1	12.1	13.0	14.7	16.5	18.7	21.7	26.3	28.8
55	4.8	6.1	8.5	10.0	11.1	12.1	13.0	14.7	16.6	18.7	21.8	26.4	28.9
56	4.8	6.1	8.5	10.0	11.1	12.1	13.0	14.7	16.6	18.8	21.8	26.5	29.0
57	4.8	6.1	8.5	10.0	11.1	12.1	13.0	14.7	16.6	18.8	21.9	26.6	29.2
58	4.8	6.0	8.4	10.0	11.1	12.1	13.0	14.8	16.7	18.9	22.0	26.7	29.3
59	4.7	6.0	8.4	10.0	11.1	12.1	13.0	14.8	16.7	18.9	22.0	26.8	29.4
60	4.7	6.0	8.4	10.0	11.1	12.1	13.0	14.8	16.7	19.0	22.1	26.9	29.5
61	4.7	6.0	8.4	10.0	11.1	12.1	13.0	14.8	16.8	19.0	22.2	27.0	29.7
62	4.6	6.0	8.4	10.0	11.1	12.1	13.0	14.8	16.8	19.1	22.2	27.1	29.8
63	4.6	5.9	8.4	10.0	11.1	12.1	13.0	14.9	16.8	19.1	22.3	27.2	29.9
64	4.6	5.9	8.4	10.0	11.1	12.1	13.0	14.9	16.9	19.2	22.4	27.4	30.0
65	4.6	5.9	8.4	10.0	11.1	12.1	13.0	14.9	16.9	19.2	22.5	27.5	30.2
66	4.5	5.9	8.4	10.0	11.1	12.1	13.0	14.9	16.9	19.3	22.5	27.6	30.3
67	4.5	5.9	8.3	10.0	11.1	12.1	13.0	14.9	17.0	19.3	22.6	27.7	30.4
68	4.5	5.8	8.3	10.0	11.1	12.1	13.1	15.0	17.0	19.4	22.7	27.8	30.5
69	4.5	5.8	8.3	9.9	11.1	12.1	13.1	15.0	17.0	19.4	22.8	27.9	30.7
70	4.4	5.8	8.3	9.9	11.1	12.1	13.1	15.0	17.1	19.5	22.8	28.0	30.8
71	4.4	5.8	8.3	9.9	11.1	12.1	13.1	15.0	17.1	19.5	22.9	28.1	30.9
72	4.4	5.8	8.3	9.9	11.1	12.1	13.1	15.0	17.1	19.6	23.0	28.2	31.1
73	4.4	5.7	8.3	9.9	11.1	12.1	13.1	15.1	17.2	19.6	23.1	28.3	31.2
74	4.3	5.7	8.3	9.9	11.1	12.1	13.1	15.1	17.2	19.7	23.1	28.5	31.3
75	4.3	5.7	8.3	9.9	11.1	12.2	13.1	15.1	17.2	19.7	23.2	28.6	31.5
76	4.3	5.7	8.3	9.9	11.1	12.2	13.1	15.1	17.3	19.8	23.3	28.7	31.6
77	4.3	5.7	8.3	9.9	11.1	12.2	13.1	15.2	17.3	19.9	23.4	28.8	31.7
78	4.3	5.7	8.2	9.9	11.1	12.2	13.1	15.2	17.4	19.9	23.4	28.9	31.8
79	4.2	5.6	8.2	9.9	11.1	12.2	13.1	15.2	17.4	20.0	23.5	29.0	32.0
80	4.2	5.6	8.2	9.9	11.1	12.2	13.2	15.2	17.4	20.0	23.6	29.1	32.1
81	4.2	5.6	8.2	9.9	11.1	12.2	13.2	15.2	17.5	20.1	23.7	29.2	32.2
82	4.2	5.6	8.2	9.9	11.1	12.2	13.2	15.3	17.5	20.1	23.7	29.3	32.4
83	4.2	5.6	8.2	9.9	11.1	12.2	13.2	15.3	17.5	20.2	23.8	29.4	32.5
84	4.1	5.6	8.2	9.9	11.1	12.2	13.2	15.3	17.6	20.2	23.9	29.5	32.6
85	4.1	5.6	8.2	9.9	11.2	12.2	13.2	15.3	17.6	20.3	24.0	29.7	32.7
86	4.1	5.5	8.2	9.9	11.2	12.2	13.2	15.3	17.6	20.3	24.0	29.8	32.9
87	4.1	5.5	8.2	9.9	11.2	12.2	13.2	15.4	17.7	20.4	24.1	29.9	33.0
88	4.1	5.5	8.2	9.9	11.2	12.2	13.2	15.4	17.7	20.4	24.2	30.0	33.1

Ages are in years; column headings are percentiles.

We show that TT peaks [mean (2.5–97.5 percentile)] at 15.4 (7.2–31.1) nmol/L at an average age of 19 years, and falls in the average case [mean (2.5–97.5 percentile)] to 13.0 (6.6–25.3) nmol/L by age 40 years, but we find no evidence for a further fall in mean TT with increasing age through to old age. However we do show that there is an increased variation in TT levels with advancing age after age 40 years. Our analyses show that the 95% prediction limit increases from 18.7 nmol/L at age 40 years to 24.5 nmol/L at age 88 years. The model provides centile and/or standard score values for an individual when compared to the population as a whole.

## Discussion

Using data-driven modelling and analysis, we have derived a normative model of total testosterone throughout the lifespan. We have shown that in the average healthy male testosterone is low in pre-puberty, rises from age 11 and peaks at age 19 at 15.4 (7.2–31.1) nmol/L [mean (2.5–97.5 percentile)]. Thereafter TT falls slightly to age 40 years to 13.0 (6.6–25.3) nmol/L. We find no evidence to support a progressive decline in testosterone in middle-aged and older men, sometimes termed the ‘andropause’, as TT does not fall significantly in the average man after the age of 40 years. Our analyses show that the 95% prediction limit increases from 18.7 nmol/L at age 40 years to 24.5 nmol/L at age 88 years. This increase in variation with increasing age demonstrates that reference ranges for TT that do not take chronological age into account are inappropriate for the assessment of an individual’s testosterone levels.

Previous studies of TT have reported contradictory results. Whilst some studies show a decline in serum TT with age throughout life, other studies report no change in testosterone levels after approximately age 35 years. In particular, from the studies used as a basis for our dataset, Travison et al. [6] observed a cross-sectional decline of 0.4% per year age from 45 years onwards in 2,194 men (95% CI of 0.2% to 0.6%), whereas Yeap et al. [29] found that TT did not decline with advancing age in older men (aged 70–89, n = 3,645). Halmenschlager et al. (n = 428) [26] report both no decline in TT with advancing years and an increase in variance later in life. Our analysis of the combined data from 13 studies shows that TT levels do not decline after age 40 years in the average case.

We now compare our results with those reported in studies that were not used to form our dataset. These comparisons are necessarily qualitative since the data were in the form of descriptive statistics or could not be reliably converted to the LC-MS/MS assay and hence were excluded during our data acquisition process. For each study we supply the number of subjects involved, n, to aid comparison with the studies used as a basis for our dataset. Rohmann et al. [27] report a decline of 1.0% per year from age 35 (n = 1,351), in qualitative disagreement to our findings. However they report geometric means as opposed to arithmetic means and do not report the sample size for each calculation, so a detailed comparison of their results to ours is not possible. Muller et al. [32], Mohr et al. [31] and Simon et al. [35] all report a small annual decline of 0.4%, 0.3% and 0.5% respectively (n = 400, 1,677, 1,408 respectively) and show no increase in variance, also in qualitative disagreement with our two key findings. Frost et al. [25], Boyce et al. [46] and Orwoll et al. [30] (n = 783, 266, 2,623 respectively) all report no decline in serum TT with advancing age but also no increase in variance. Rhoden et al. [34] report that not only does serum TT not fall with advancing age, but there is also an increase in variance across the lifespan from age 40 onwards (n = 1,071). Taken together, these studies provide partial qualitative external validation for our model, but do not completely resolve the issue of contradictory single-centre study outcomes.

Our model is derived from data from multiple sources of the measurement of TT in over 10,000 healthy males aged between 3 and 101 years. This is both a strength and weakness of the study. The strength is that modelling power is increased by the provision of large numbers of datapoints for a wide range of ages: it has been previously shown that models that include both prepubertal, pubertal and adult ages can be used to derive important insights for a restricted age range [47]. The weakness is the approximate heterogeneity of the values obtained from diverse sources, especially as assay conversion factors were used that have known high correlation but are nevertheless inexact. This includes studies that involve convenience samples (e.g. primary care and out-patient attenders) as well as those that involve population derived cohorts. Further limitations of our approach are that insufficient data were found to model accurately neonatal ages, and that we had to exclude potentially useful studies that used in-house assays which lack standardisation and harmonisation, and for which no conversion formula has been published [48]. Ten of the thirteen studies used as data sources (Table 1) excluded subjects taking medication that could affect the endocrine system, but three studies [5], [6], [49] (combined n = 2,371 of 10,097) do not have equivalently explicit inclusion criteria. We can therefore not rule out the possibility that a small number of subjects were on medication that increased their TT levels.

Our results suggest that the reported increase in the proportion of hypogonadal men with increasing age can be attributed to the increase in variance of testosterone levels with increasing age, as opposed to an age-related decline in testosterone levels for the population as a whole. In particular, our model provides a coherent explanation for the widely believed but incorrect assertion that the prevalence of male hypogonadism increases from 12% in men in their 50s to 49% in men in their 80s when hypogonadism is defined as a TT level lower than the 2.5 percentile [5], [50], or lower than 6.4 nmol/L using the LC-MS/MS assay. These assertions are incorrect since it is not possible to have more than 2.5% of the population included in an age-related 2.5^th^ centile. However, if the definition of hypogonadism is based on a TT level lower than a fixed value, then the prevalence of hypogonadism will indeed increase due to increased variance with advancing age. A common rationale for the increased prevalence of low serum testosterone levels is the assertion of an annual average case decline of 1% or more [6], [50]. As shown above, the majority of cross-sectional studies either report no decline in the average case, or a moderate annual decline in testosterone levels. Again, the disparity between the common explanation given and the data in the literature is that a greater proportion of individuals have lower levels of testosterone with increasing age.

There is disagreement on the indications for the use of hormonal therapy in men with apparently age-related low testosterone concentrations [51], [52]. Our analysis of the combined data from several studies agrees to a certain extent with both sides of the controversy. We find no evidence that TT declines in the average case after the age of 40 years for ageing males. We do find that the prevalence of higher and lower testosterone levels increases with age, and hence that there is a larger number of men potentially at risk of androgen-related disorders. Our study shows that the increasing proportion of men commonly regarded as having abnormally low (or high) levels of TT can be accounted for by the increase in variance in testosterone levels with age. Factors that have been identfied as determinants of lower testosterone include obesity in many studies, as well as the development of other co-morbidities [53]–[55]. Obesity was not an exclusion criteria for our data acquistion, and our age-related reference ranges can be used for quantitative evaluation of the relationship between high body-mass index and low testosterone. There is increasing concern over testosterone supplementation in men, both generally and more particulary in those with comorbidities [20], [21]. Whether all or subgroups of older hypogonadal men might benefit from replacement requires critical assessment, for which a robustly-established normal range provides an important basis.

This analysis also highlights the increasing proportion of men with high testosterone with increasing age. This intriguing finding is both at odds with the notion of an age-related fall in the general case, and may be relevant to diseases more prevalent in this age group. For example, a recent study has shown an association in older men between high testosterone and increased all-cause mortality when compared to those with mid-range testosterone levels [56], although causality or indeed reverse causality are not established.

In conclusion, This model provides the reference ranges needed to support research and clinical decision making in males who have symptoms that may be due to hypogonadism. In addition, our study suggests that instead of a gradual decline in testosterone levels in men as they age there is an increasing variation in testosterone levels in aged men with a larger population of hypogonadal males, who may benefit from testosterone therapy, but also more men with high serum total testosterone that may also be disadvantageous [56].

## Supporting Information

Figure S1 **Figure S1a.** Model residuals for ages 3 through 11 years. The residuals are the variations in log-adjusted observed values from the log-adjusted age-related mean value predicted by the model. **Figure S1b.** Model residuals for ages 20 through 29 years. The residuals are the variations in log-adjusted observed values from the log-adjusted age-related mean value predicted by the model. **Figure S1c.** Model residuals for ages 30 through 39 years. The residuals are the variations in log-adjusted observed values from the log-adjusted age-related mean value predicted by the model. **Figure S1d.** Model residuals for ages 40 through 49 years. The residuals are the variations in log-adjusted observed values from the log-adjusted age-related mean value predicted by the model. **Figure S1e.** Model residuals for ages 50 through 59 years. The residuals are the variations in log-adjusted observed values from the log-adjusted age-related mean value predicted by the model. **Figure S1f.** Model residuals for ages 60 through 69 years. The residuals are the variations in log-adjusted observed values from the log-adjusted age-related mean value predicted by the model. **Figure S1g.** Model residuals for ages 70 through 79 years. The residuals are the variations in log-adjusted observed values from the log-adjusted age-related mean value predicted by the model. **Figure S1h.** Model residuals for ages 80 through 89 years. The residuals are the variations in log-adjusted observed values from the log-adjusted age-related mean value predicted by the model.
(DOCX)

Figure S2 **Model validation.** An exemplar of the 5-fold cross validation analysis is given as Figure 3 of the main text; this figure shows the remaining four cases. High test and training errors represent underfit (i.e. insufficient model parameters to accurately capture essential features of the dataset), and high test errors represent overfit (i.e. a model that will not generalise to accurately predict new data). An optimal number of model parameters is seven in all cases.
(TIFF)

Table S1 **TableCurve inputs and output for the validated model.**
(XLSX)
