# Correction: A Validated Age-Related Normative Model for Male Total Testosterone Shows Increasing Variance but No Decline after Age 40 Years

**DOI:** 10.1371/journal.pone.0117674

**Published:** 2015-02-06

**Authors:** The PLOS ONE Staff

There are errors in Table 3. Please view the corrected Table 3 here.

**Table 3:** Normative age-related total testosterone reference values in nmol/L. Ages are in years; column headings are percentiles.

**Age**	**1**	**2.5**	**10**	**20**	**30**	**40**	**50**	**60**	**70**	**80**	**90**	**97.5**	**99**
3	0.0	0.0	0.0	0.1	0.2	0.2	0.4	0.6	0.6	0.7	0.8	0.9	1.0
4	0.0	0.0	0.1	0.1	0.2	0.2	0.4	0.6	0.6	0.7	0.8	0.9	1.0
5	0.0	0.0	0.1	0.1	0.2	0.2	0.4	0.6	0.6	0.7	0.8	0.8	1.0
6	0.0	0.0	0.0	0.1	0.1	0.2	0.3	0.5	0.6	0.6	0.7	0.8	0.9
7	0.0	0.0	0.0	0.0	0.1	0.1	0.3	0.5	0.5	0.6	0.7	0.7	0.9
8	0.0	0.0	0.0	0.0	0.0	0.1	0.2	0.4	0.5	0.5	0.6	0.7	0.9
9	0.0	0.0	0.0	0.0	0.0	0.0	0.2	0.5	0.5	0.6	0.7	0.8	1.0
10	0.0	0.0	0.0	0.0	0.0	0.1	0.3	0.7	0.8	0.9	1.0	1.2	1.4
11	0.0	0.0	0.0	0.2	0.3	0.3	0.7	1.3	1.4	1.6	1.9	2.1	2.6
12	0.0	0.3	0.5	0.8	1.0	1.1	1.7	2.8	3.1	3.4	3.9	4.3	5.2
13	0.6	1.4	1.7	2.2	2.5	2.7	3.8	5.9	6.4	7.0	8.0	8.8	10.3
14	1.8	3.0	3.6	4.4	4.8	5.2	6.9	10.5	11.4	12.4	14.0	15.3	18.0
15	2.9	4.6	5.6	6.6	7.3	7.9	10.3	15.4	16.5	18.0	20.2	22.1	25.9
16	3.7	5.9	7.0	8.3	9.2	9.8	12.9	19.0	20.4	22.2	24.9	27.2	31.7
17	4.1	6.6	7.8	9.3	10.3	11.1	14.4	21.1	22.6	24.6	27.5	30.0	34.9
18	4.4	7.0	8.3	9.8	10.8	11.7	15.2	22.0	23.6	25.6	28.5	31.1	36.1
19	4.6	7.2	8.5	10.0	11.1	11.9	15.4	22.2	23.7	25.7	28.6	31.1	36.1
20	4.7	7.3	8.6	10.1	11.1	11.9	15.4	21.9	23.5	25.4	28.2	30.7	35.5
21	4.8	7.3	8.6	10.0	11.0	11.8	15.2	21.6	23.0	24.9	27.6	30.0	34.7
22	4.9	7.3	8.5	10.0	10.9	11.7	15.0	21.2	22.6	24.4	27.0	29.3	33.8
23	4.9	7.3	8.5	9.9	10.8	11.5	14.8	20.7	22.1	23.9	26.4	28.7	33.1
24	4.9	7.2	8.4	9.8	10.7	11.4	14.5	20.4	21.7	23.4	25.9	28.1	32.3
25	4.9	7.2	8.3	9.7	10.6	11.3	14.3	20.0	21.3	23.0	25.4	27.6	31.7
26	5.0	7.2	8.3	9.6	10.4	11.1	14.1	19.7	21.0	22.6	25.0	27.1	31.2
27	4.9	7.1	8.2	9.5	10.3	11.0	14.0	19.5	20.7	22.3	24.7	26.7	30.7
28	4.9	7.1	8.2	9.4	10.3	10.9	13.8	19.2	20.5	22.1	24.4	26.4	30.4
29	4.9	7.0	8.1	9.3	10.2	10.8	13.7	19.1	20.3	21.8	24.2	26.2	30.1
30	4.9	7.0	8.0	9.3	10.1	10.8	13.6	18.9	20.1	21.7	24.0	25.9	29.8
31	4.9	6.9	8.0	9.2	10.0	10.7	13.5	18.8	20.0	21.5	23.8	25.8	29.6
32	4.8	6.9	7.9	9.1	10.0	10.6	13.4	18.7	19.9	21.4	23.7	25.6	29.4
33	4.8	6.8	7.9	9.1	9.9	10.6	13.3	18.6	19.8	21.3	23.6	25.5	29.3
34	4.8	6.8	7.8	9.0	9.9	10.5	13.3	18.5	19.7	21.2	23.5	25.4	29.2
35	4.7	6.8	7.8	9.0	9.8	10.5	13.2	18.4	19.6	21.2	23.4	25.4	29.2
36	4.7	6.7	7.8	9.0	9.8	10.4	13.2	18.4	19.6	21.1	23.4	25.3	29.1
37	4.7	6.7	7.7	8.9	9.7	10.4	13.1	18.4	19.6	21.1	23.3	25.3	29.1
38	4.6	6.7	7.7	8.9	9.7	10.3	13.1	18.3	19.5	21.1	23.3	25.3	29.1
39	4.6	6.6	7.7	8.9	9.7	10.3	13.1	18.3	19.5	21.1	23.3	25.3	29.1
40	4.5	6.6	7.6	8.8	9.6	10.3	13.0	18.3	19.5	21.1	23.3	25.3	29.2
41	4.5	6.5	7.6	8.8	9.6	10.2	13.0	18.3	19.5	21.1	23.4	25.3	29.2
42	4.5	6.5	7.6	8.8	9.6	10.2	13.0	18.3	19.6	21.1	23.4	25.4	29.3
43	4.4	6.5	7.5	8.7	9.5	10.2	13.0	18.3	19.6	21.1	23.4	25.4	29.3
44	4.4	6.4	7.5	8.7	9.5	10.2	13.0	18.4	19.6	21.2	23.5	25.5	29.4
45	4.4	6.4	7.5	8.7	9.5	10.2	13.0	18.4	19.6	21.2	23.5	25.6	29.5
46	4.3	6.4	7.4	8.7	9.5	10.1	13.0	18.4	19.7	21.2	23.6	25.6	29.6
47	4.3	6.3	7.4	8.6	9.5	10.1	13.0	18.4	19.7	21.3	23.6	25.7	29.7
48	4.2	6.3	7.4	8.6	9.4	10.1	13.0	18.5	19.7	21.3	23.7	25.8	29.8
49	4.2	6.3	7.4	8.6	9.4	10.1	13.0	18.5	19.8	21.4	23.8	25.9	29.9
50	4.2	6.3	7.3	8.6	9.4	10.1	13.0	18.5	19.8	21.4	23.9	25.9	30.0
51	4.1	6.2	7.3	8.6	9.4	10.1	13.0	18.6	19.9	21.5	23.9	26.0	30.1
52	4.1	6.2	7.3	8.5	9.4	10.1	13.0	18.6	19.9	21.6	24.0	26.1	30.3
53	4.0	6.2	7.3	8.5	9.4	10.0	13.0	18.6	20.0	21.6	24.1	26.2	30.4
54	4.0	6.2	7.2	8.5	9.4	10.0	13.0	18.7	20.0	21.7	24.2	26.3	30.5
55	4.0	6.1	7.2	8.5	9.3	10.0	13.0	18.7	20.1	21.8	24.2	26.4	30.6
56	3.9	6.1	7.2	8.5	9.3	10.0	13.0	18.8	20.1	21.8	24.3	26.5	30.8
57	3.9	6.1	7.2	8.5	9.3	10.0	13.0	18.8	20.2	21.9	24.4	26.6	30.9
58	3.9	6.0	7.2	8.4	9.3	10.0	13.0	18.9	20.2	22.0	24.5	26.7	31.0
59	3.8	6.0	7.1	8.4	9.3	10.0	13.0	18.9	20.3	22.0	24.6	26.8	31.2
60	3.8	6.0	7.1	8.4	9.3	10.0	13.0	19.0	20.4	22.1	24.7	26.9	31.3
61	3.8	6.0	7.1	8.4	9.3	10.0	13.0	19.0	20.4	22.2	24.8	27.0	31.4
62	3.7	6.0	7.1	8.4	9.3	10.0	13.0	19.1	20.5	22.2	24.9	27.1	31.6
63	3.7	5.9	7.1	8.4	9.3	10.0	13.0	19.1	20.5	22.3	25.0	27.2	31.7
64	3.7	5.9	7.1	8.4	9.3	10.0	13.0	19.2	20.6	22.4	25.1	27.4	31.9
65	3.7	5.9	7.0	8.4	9.3	10.0	13.0	19.2	20.7	22.5	25.1	27.5	32.0
66	3.6	5.9	7.0	8.4	9.2	10.0	13.0	19.3	20.7	22.5	25.2	27.6	32.1
67	3.6	5.9	7.0	8.3	9.2	10.0	13.0	19.3	20.8	22.6	25.3	27.7	32.3
68	3.6	5.8	7.0	8.3	9.2	10.0	13.1	19.4	20.8	22.7	25.4	27.8	32.4
69	3.5	5.8	7.0	8.3	9.2	9.9	13.1	19.4	20.9	22.8	25.5	27.9	32.6
70	3.5	5.8	7.0	8.3	9.2	9.9	13.1	19.5	21.0	22.8	25.6	28.0	32.7
71	3.5	5.8	6.9	8.3	9.2	9.9	13.1	19.5	21.0	22.9	25.7	28.1	32.9
72	3.5	5.8	6.9	8.3	9.2	9.9	13.1	19.6	21.1	23.0	25.8	28.2	33.0
73	3.4	5.7	6.9	8.3	9.2	9.9	13.1	19.6	21.2	23.1	25.9	28.3	33.1
74	3.4	5.7	6.9	8.3	9.2	9.9	13.1	19.7	21.2	23.1	26.0	28.5	33.3
75	3.4	5.7	6.9	8.3	9.2	9.9	13.1	19.7	21.3	23.2	26.1	28.6	33.4
76	3.4	5.7	6.9	8.3	9.2	9.9	13.1	19.8	21.3	23.3	26.2	28.7	33.6
77	3.3	5.7	6.9	8.3	9.2	9.9	13.1	19.9	21.4	23.4	26.3	28.8	33.7
78	3.3	5.7	6.9	8.2	9.2	9.9	13.1	19.9	21.5	23.4	26.4	28.9	33.8
79	3.3	5.6	6.8	8.2	9.2	9.9	13.1	20.0	21.5	23.5	26.5	29.0	34.0
80	3.3	5.6	6.8	8.2	9.2	9.9	13.2	20.0	21.6	23.6	26.5	29.1	34.1
81	3.2	5.6	6.8	8.2	9.2	9.9	13.2	20.1	21.7	23.7	26.6	29.2	34.3
82	3.2	5.6	6.8	8.2	9.2	9.9	13.2	20.1	21.7	23.7	26.7	29.3	34.4
83	3.2	5.6	6.8	8.2	9.2	9.9	13.2	20.2	21.8	23.8	26.8	29.4	34.5
84	3.2	5.6	6.8	8.2	9.2	9.9	13.2	20.2	21.8	23.9	26.9	29.5	34.7
85	3.1	5.6	6.8	8.2	9.2	9.9	13.2	20.3	21.9	24.0	27.0	29.7	34.8
86	3.1	5.5	6.8	8.2	9.2	9.9	13.2	20.3	22.0	24.0	27.1	29.8	35.0
87	3.1	5.5	6.8	8.2	9.1	9.9	13.2	20.4	22.0	24.1	27.2	29.9	35.1
88	3.1	5.5	6.8	8.2	9.1	9.9	13.2	20.4	22.1	24.2	27.3	30.0	35.2

There are errors in Fig. 5 and the legend for Fig. 5. Please view the corrected Fig. 5 and the complete, correct Fig. 5 legend here.

**Fig 5:**
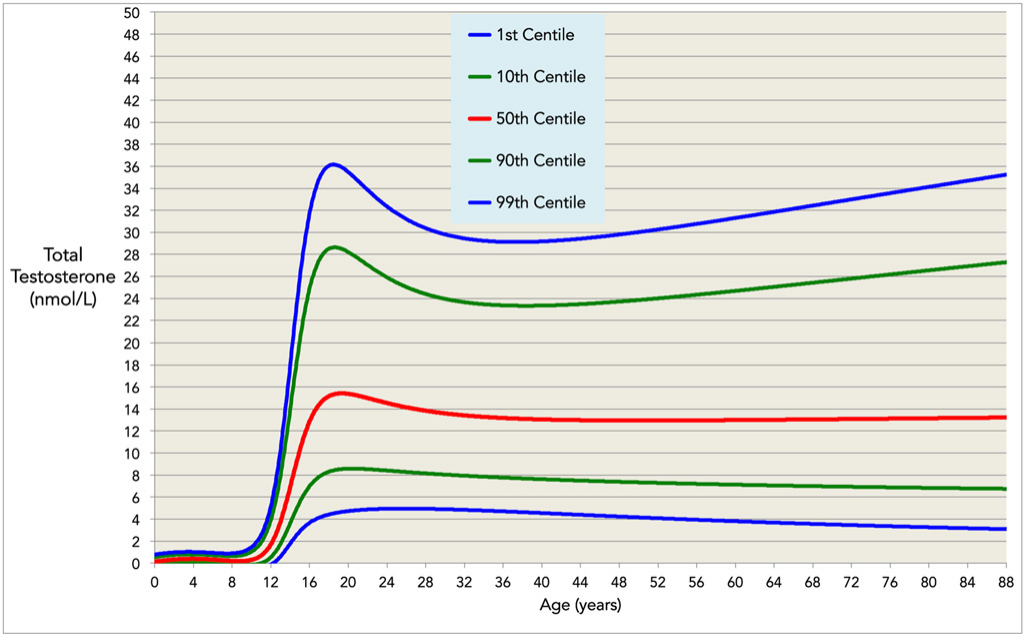
The validated model in centiles. Normative ranges for the model of total testosterone from ages 3–88 years. In the average case (red line) total testosterone remains constant for age > 40. However, the variance in normative ranges increases for these ages, with 1^st^ to 99^th^ centile ranges of 4.7–29.2 nmol/L at age 35 years and 3.1–35.2 nmol/L at age 88 years.
