# Supplementary material for: A Validated Age-Related Normative Model for Male Total Testosterone Shows Increasing Variance but No Decline after Age 40 Years
Source: PLoS One. 2014 Oct 8;9(10):e109346. doi: 10.1371/journal.pone.0109346 (PMC4190174; doi:10.1371/journal.pone.0109346)
Supplement: Figure S1 — a. Model residuals for ages 3 through 11 years. The residuals are the variations in log-adjusted observed values from the log-adjusted age-related mean value predicted by the model. Figure S1b. Model residuals for ages 20 through 29 years. The residuals are the variations in log-adjusted observed values from the log-adjusted age-related mean value predicted by the model. Figure S1c. Model residuals for ages 30 through 39 years. The residuals are the variations in log-adjusted observed values from the log-adjusted age-related mean value predicted by the model. Figure S1d. Model residuals for ages 40 through 49 years. The residuals are the variations in log-adjusted observed values from the log-adjusted age-related mean value predicted by the model. Figure S1e. Model residuals for ages 50 through 59 years. The residuals are the variations in log-adjusted observed values from the log-adjusted age-related mean value predicted by the model. Figure S1f. Model residuals for ages 60 through 69 years. The residuals are the variations in log-adjusted observed values from the log-adjusted age-related mean value predicted by the model. Figure S1g. Model residuals for ages 70 through 79 years. The residuals are the variations in log-adjusted observed values from the log-adjusted age-related mean value predicted by the model. Figure S1h. Model residuals for ages 80 through 89 years. The residuals are the variations in log-adjusted observed values from the log-adjusted age-related mean value predicted by the model. (DOCX) [file pone.0109346.s001.docx]

**A validated age-related normative model for male total testosterone shows increasing variance but no decline after age 40 years.**

**Supplementary Information 1.** Residual plots for age ranges.

**
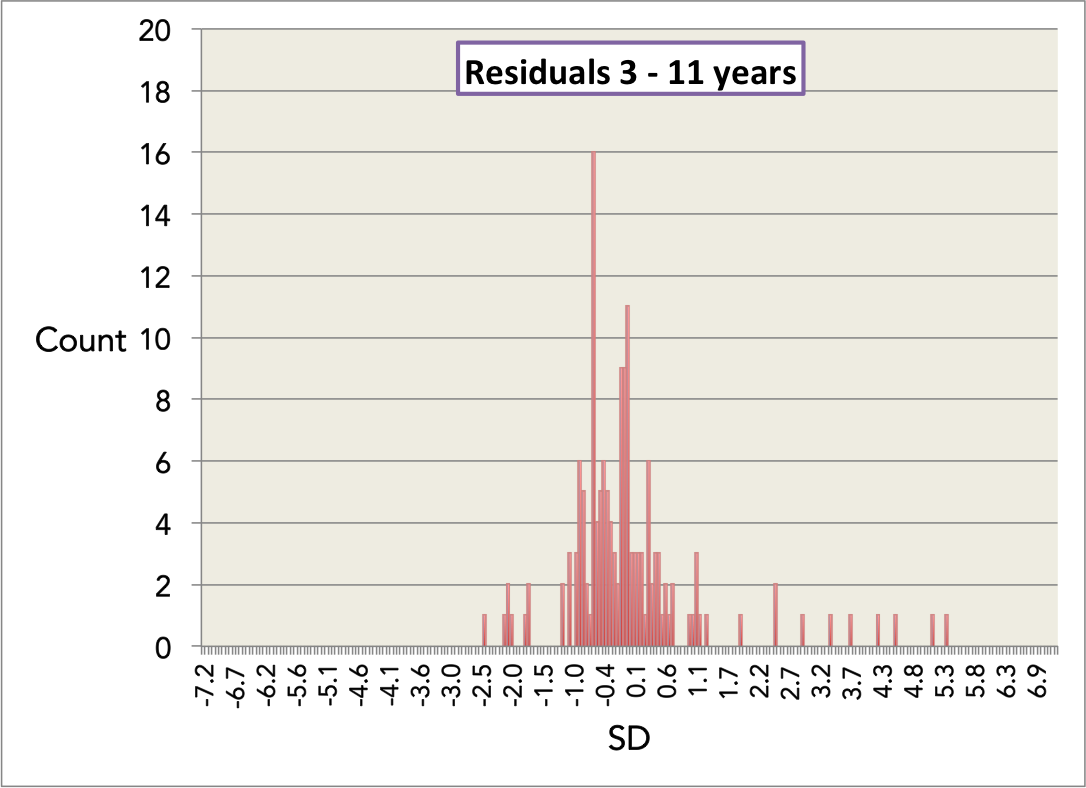
**

**
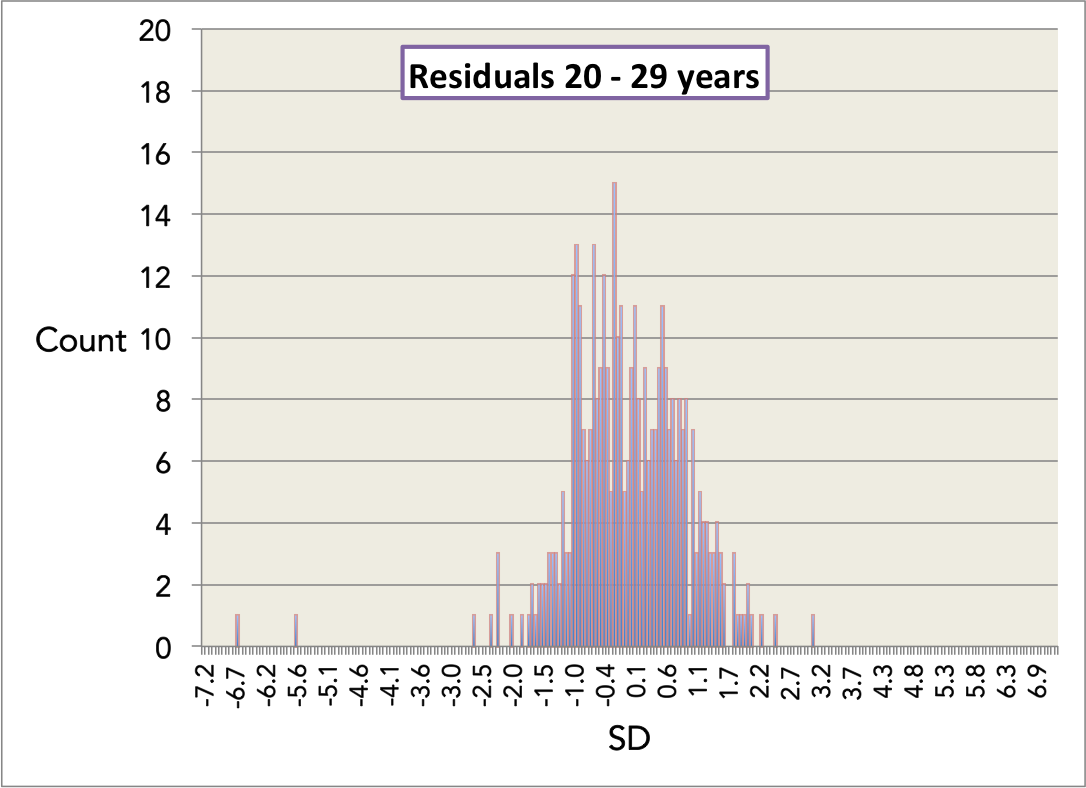
**

**
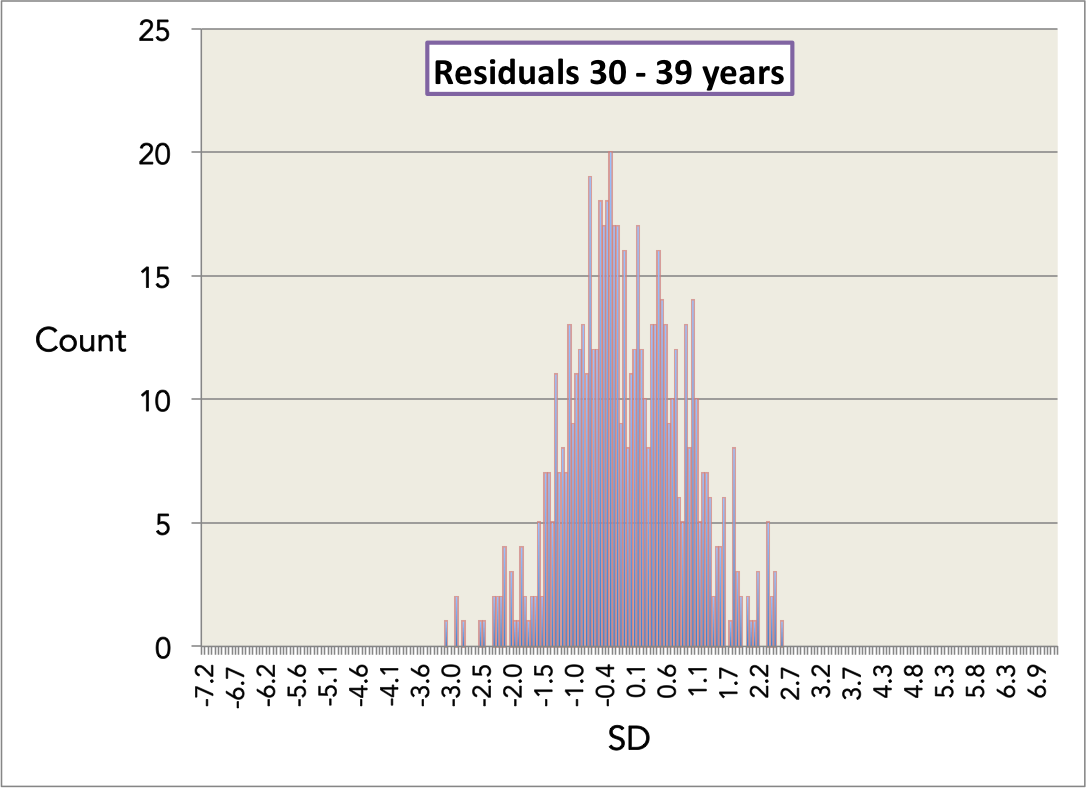
**

**
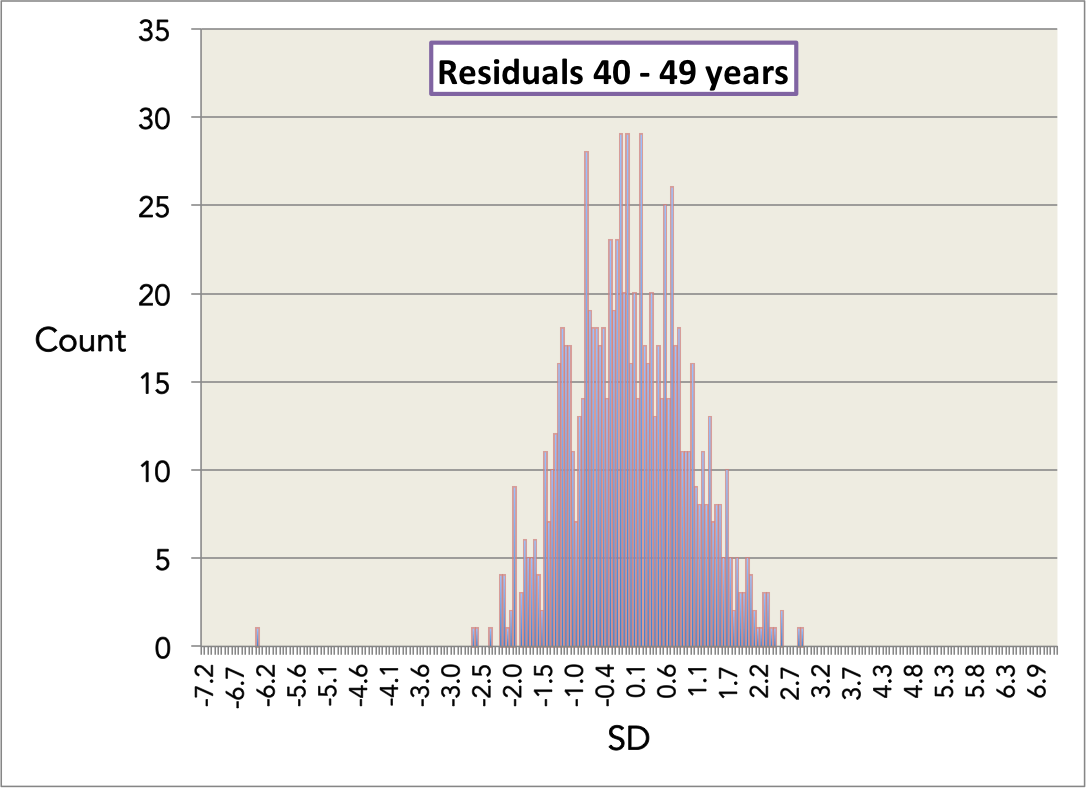
**

**
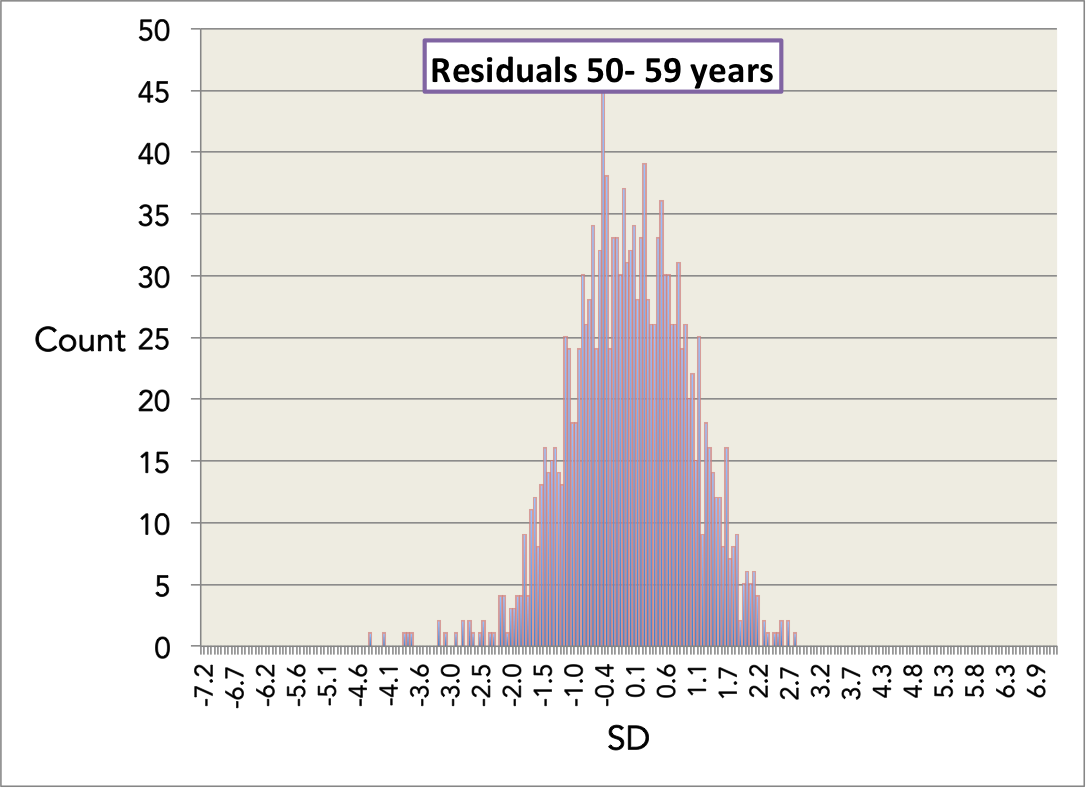
**

**
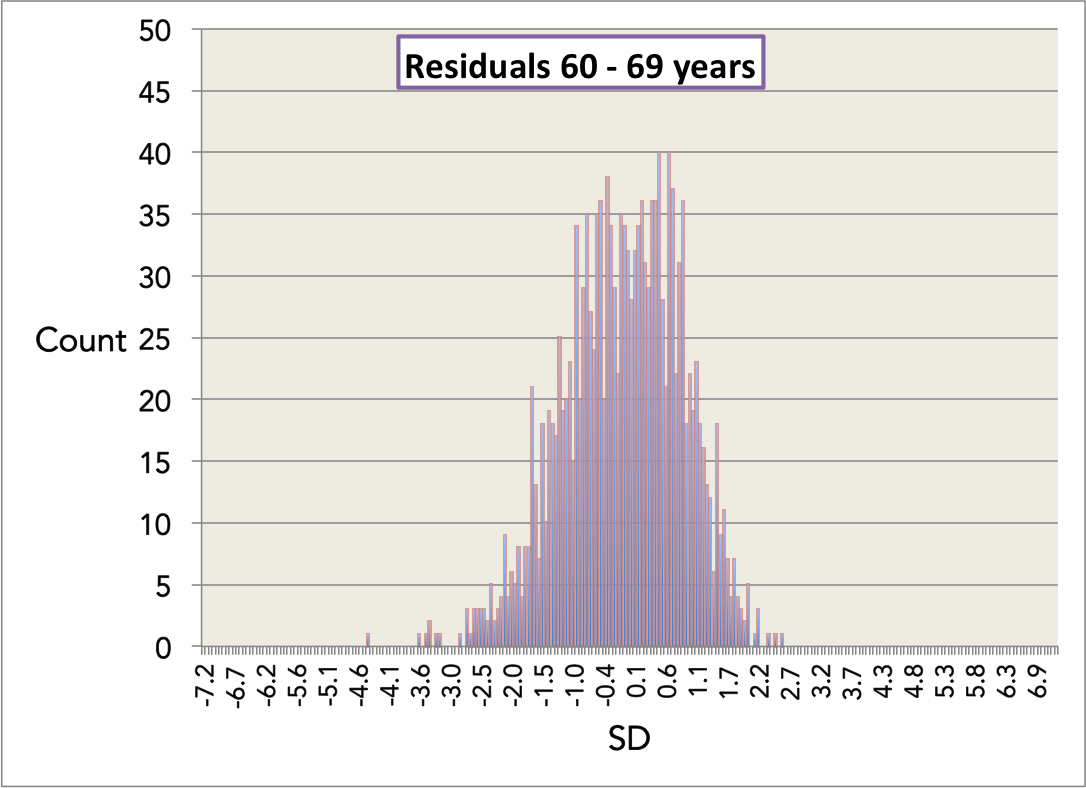
**

**
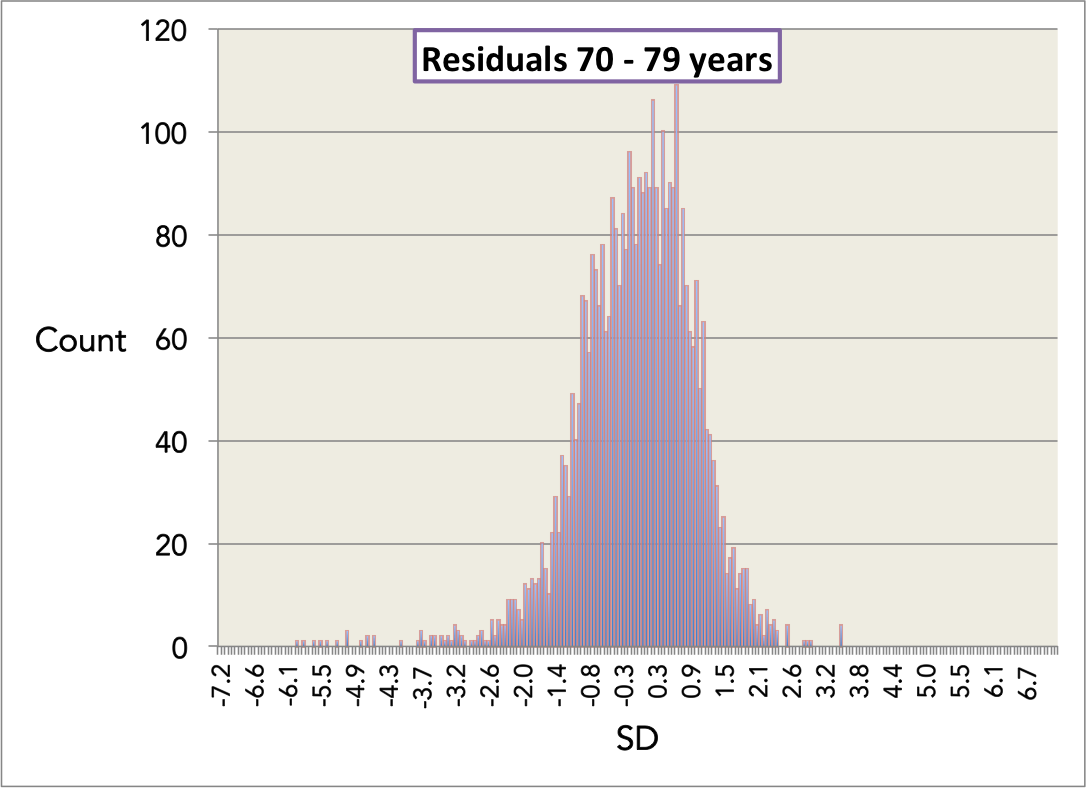
**

**
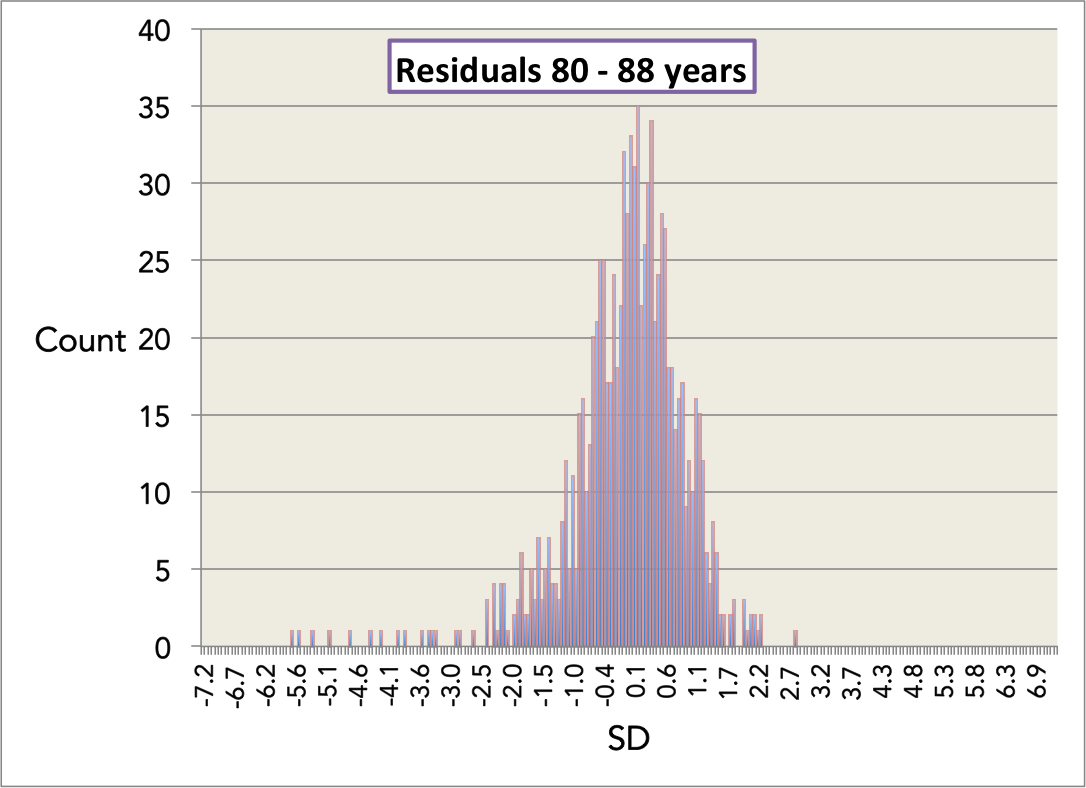
**
