# Supplementary figures and images for: A Validated Age-Related Normative Model for Male Total Testosterone Shows Increasing Variance but No Decline after Age 40 Years
Source: PLoS One. 2014 Oct 8;9(10):e109346. doi: 10.1371/journal.pone.0109346 (PMC4190174; doi:10.1371/journal.pone.0109346)

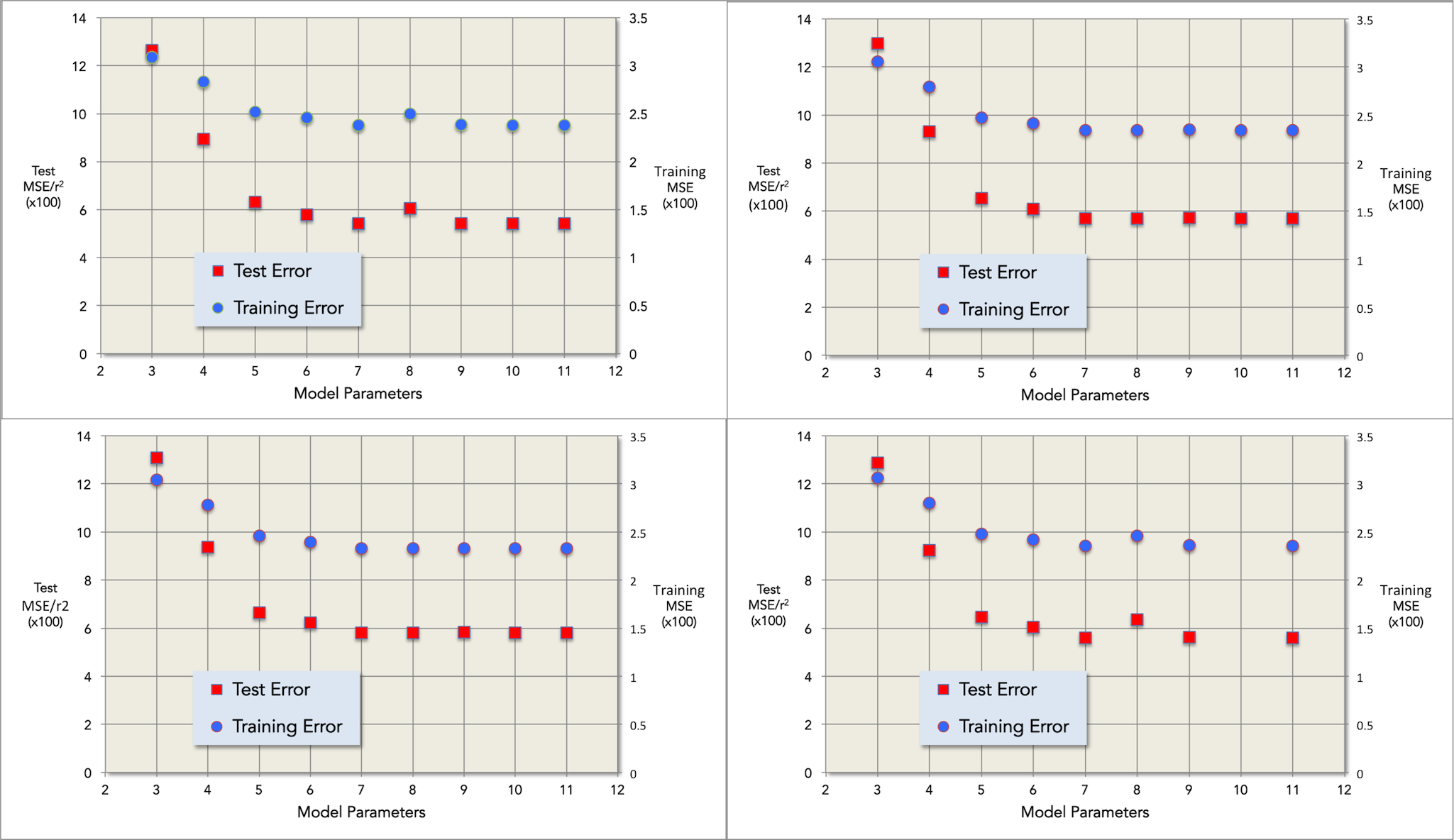

Supplement: Figure S2 — Model validation. An exemplar of the 5-fold cross validation analysis is given as Figure 3 of the main text; this figure shows the remaining four cases. High test and training errors represent underfit (i.e. insufficient model parameters to accurately capture essential features of the dataset), and high test errors represent overfit (i.e. a model that will not generalise to accurately predict new data). An optimal number of model parameters is seven in all cases. (TIFF) [file pone.0109346.s002.tiff]
